# Novel *de novo BRCA2 *mutation in a patient with a family history of breast cancer

**DOI:** 10.1186/1471-2350-9-58

**Published:** 2008-07-02

**Authors:** Thomas V O Hansen, Marie Luise Bisgaard, Lars Jønson, Anders Albrechtsen, Bettina Filtenborg-Barnkob, Hans Eiberg, Bent Ejlertsen, Finn C Nielsen

**Affiliations:** 1 Department of Clinical Biochemistry, Rigshospitalet, Copenhagen, Denmark; 2 Department of Cellular and Molecular Medicine, Panum Institute, University of Copenhagen, Denmark; 3 Department of Biostatistics, University of Copenhagen, Copenhagen, Denmark; 4 Department of Pathology, Roskilde Hospital, Roskilde, Denmark; 5 Department of Oncology, Rigshospitalet, Copenhagen, Denmark

## Abstract

**Background:**

*BRCA2 *germ-line mutations predispose to breast and ovarian cancer. Mutations are widespread and unclassified splice variants are frequently encountered. We describe the parental origin and functional characterization of a novel *de novo BRCA2 *splice site mutation found in a patient exhibiting a ductal carcinoma at the age of 40.

**Methods:**

Variations were identified by denaturing high performance liquid chromatography (dHPLC) and sequencing of the *BRCA1 *and *BRCA2 *genes. The effect of the mutation on splicing was examined by exon trapping in COS-7 cells and by RT-PCR on RNA isolated from whole blood. The paternity was determined by single nucleotide polymorphism (SNP) microarray analysis. Parental origin of the *de novo *mutation was determined by establishing mutation-SNP haplotypes by variant specific PCR, while *de novo *and mosaic status was investigated by sequencing of DNA from leucocytes and carcinoma tissue.

**Results:**

A novel *BRCA2 *variant in the splice donor site of exon 21 (nucleotide 8982+1 G→A/c.8754+1 G→A) was identified. Exon trapping showed that the mutation activates a cryptic splice site 46 base pairs 3' of exon 21, resulting in the inclusion of a premature stop codon and synthesis of a truncated BRCA2 protein. The aberrant splicing was verified by RT-PCR analysis on RNA isolated from whole blood of the affected patient. The mutation was not found in any of the patient's parents or in the mother's carcinoma, showing it is a *de novo *mutation. Variant specific PCR indicates that the mutation arose in the male germ-line.

**Conclusion:**

We conclude that the novel *BRCA2 *splice variant is a *de novo *mutation introduced in the male spermatozoa that can be classified as a disease causing mutation.

## Background

Germ-line *BRCA2 *(MIM# 600185) mutations in female carriers confer a lifetime risk exceeding 80% for breast cancer and 20% for ovarian cancer, and a moderate increased risk of other cancer types [1,2]. Studies have indicated that the risk of ovarian cancer is greatest in women with *BRCA2 *mutations localized in nucleotides 3035–6629 [3,4]. Several genes are likely to be involved in breast cancer predisposition, but no susceptibility gene aside *BRCA2 *and *BRCA1 *is likely to account for a large fraction or a major increase in risk [5].

The *BRCA2 *gene spans approximately 70 kb and is composed of 27 exons, which encodes a protein of 3418 amino acids. BRCA2 is involved in homologous recombination [6,7], but is also suggested to play a role in transcriptional regulation [8] and cell cycle control [9]. Mutations are distributed throughout the entire coding region of *BRCA2 *and to date numerous deleterious mutations have been reported (Breast Cancer Information Core [BIC]; [10]). The majority of patients with *BRCA1 *or *BRCA2 *associated breast and/or ovarian cancer have a family history, although healthy male carriers may obscure the apparent dominant trait. Several *BRCA2 *founder mutations have been identified, including the Ashkenazi Jewish nucleotide 6174delT mutation and the Icelandic nucleotide 999del5 mutation [11,12]. In contrast, only two mutations in *BRCA2 *and one in *BRCA1 *have been reported as *de novo *mutations [13-15], but since a positive family history is one of the criteria for mutation screening, it is possible that we overlook a number of patients with these mutations.

Here we report the functional characterization of a novel *de novo BRCA2 *splice site mutation located in the intervening sequence (IVS) of exon 21 (nucleotide 8982+1 G→A/c.8754+1 G→A) in a Danish breast cancer patient with a family history of breast cancer. The parental origin of the mutation is assigned to the father.

## Methods

### Patients

Family B49363 is a 23 member, three-generation kindred with two affected subjects. A 40 year old woman was referred to genetic counseling two months after she had breast-conserving surgery at Roskilde County Hospital with radical excision of an 8 mm large invasive ductal carcinoma. The tumor was estrogen and progesterone receptor positive and had malignancy grade II. The patient received adjuvant radiotherapy followed by seven series of chemotherapy with CEF and tamoxifen. The patient's mother had a mastectomy at age 59 with excision of a 22 mm large estrogen and progesterone receptor positive invasive ductal carcinoma, malignancy grade II, and postoperatively she received tamoxifen. Following verbal and written consent, blood and tumour samples were collected from the proband for mutation screening and after renewed consent a third blood sample was collected for RNA analysis. Moreover, blood and tumour samples from the patient's mother, and blood samples from the patient's father and sister were collected. The family history was verified using the registry of the DBCG (Danish Breast Cancer Cooperative Group), hospital medical records and pathology reports, and genetic counselling was provided for each family member. Since the study is part of normal diagnostic procedures no ethical approval was obtained. The study was conducted in accordance with the Helsinki Declaration.

### BRCA1 and BRCA2 screening

Genomic DNA was purified from whole blood using the QIAamp DNA mini kit (Qiagen) according to the manufacturer's instructions. *BRCA1 *and *BRCA2 *were amplified using intronic primer pairs flanking each exon. PCR products were pre-screened by dHPLC (denaturing high performance liquid chromatography) using the WAWE system (Transgenomic) and sequenced using an ABI3730 DNA analyzer (Applied Biosystems). Sequence variations were verified in a new blood sample. Moreover genomic DNA was examined by MLPA analysis (MRC-Holland). The *BRCA1 *variant is numbered according to GenBank accession number U14680, in which the A in the AUG start codon has number 120, whereas the *BRCA2 *variant is numbered according to GenBank accession number NC_000059, in which A in the AUG start codon has number 229. Moreover, the *BRCA1 *and *BRCA2 *variants are numbered according to GenBank accession number NC_000017.9 and NC_000013.9 using the guidelines from the Human Genetic Variation Society [16].

### Vector constructs

The pSPL3 vector was obtained from Gibco-BRL. pSPL3-BRCA2-wild-type and pSPL3-BRCA2-mutant plasmids, containing BRCA2 exon 21 and flanking intron sequences, were constructed by PCR using purified DNA from human blood samples and the following oligonucleotides: BRCA2-F, 5'-GATCACGAATTCTTCCTGGAAAACTTATAGCA-3' and BRCA2-R 5'-GATCACCTCGAGTTAGGGTAGAGGATTATCAAGTACA-3'. The PCR products were treated with EcoRI and XhoI and cloned into the pSPL3 vector. All constructs were verified by sequencing.

### Cell culture and transfections

COS-7 cells were cultured in Dulbecco's modified Eagle's medium (DMEM) containing 4500 mg/l glucose supplemented with 10% foetal bovine serum, 100 U of penicillin per ml, and 100 μg of streptomycin per ml at 5% CO_2 _and 37°C. One day before transfection, cells were seeded in 6-well culture dishes at a density of 4 × 10^5 ^cells/well. Cells were transfected with a total of 4 μg plasmid DNA using FuGENE 6 transfection reagent (Roche) according to the manufacturer's instructions. Transfection efficiencies for each series were determined by cotransfection of pEGFP plasmid (Clontech). The next day the media was changed and after another 24 h the cells were harvested and total RNA was isolated using Trizol reagent (Invitrogen).

### Exon trapping analysis

First strand cDNA was synthesized using 1 μg RNA, 20 μM SA2 primer (5'-ATCTCAGTGGTATTTGTGAGC-3') and M-MuLV reverse transcriptase (New England Biolabs). The cDNAs were amplified with pSPL3 vector-specific primers (5'-TCTGAGTCACCTGGACAACC-3' and 5'-ATCTCAGTGGTATTTGTGAGC-3') and the PCR products were resolved on a 2% agarose gel. All experiments were repeated three times.

### RNA analysis

A fresh blood sample was obtained from the patient. Total cellular RNA was isolated with Trizol (Invitrogen) according to the manufacturer's instructions. For reverse transcription-PCR (RT-PCR), cDNA was synthesized using the AMV reverse transcriptase (Promega) as described by the supplier. The cDNA were amplified with the *BRCA2 *specific primers 5'-CGGCCTGCTCGCTGGTAT-3' and 5'-GCCTTCCTAATTTCCAACTGGATCTG-3' resulting in a 503 bp fragment. The samples were separated by agarose gel electrophoresis and visualized by ethidium bromide staining. Finally, the bands were purified, cloned into pCR-Blunt II-TOPO (Invitrogen) and sequenced using an ABI3730 DNA analyzer (Applied Biosystems).

### SNP chip analysis

Genomic DNA was applied to 250K *Sty*I (~238.000 SNPs) SNP-microarray chips and processed according to the manufacturer's instructions (Affymetrix, UK). Briefly, 250 ng of genomic DNA was digested with *StyI *and ligated to adapters. Adapter-ligated DNA was amplified, purified, fragmented and labeled with biotin and hybridized to the arrays for 18 h. The Affymetrix 450 fluidics station and the Affymetrix 3000 G7 gene scanner were used to wash, stain, and scan the arrays. The CEL files were analysed using the BRLMM algorithm from Affymetrix Genotyping analysis software (GTYPE). Relationship was inferred by estimating the pairwise identical by descent (IBD) sharing, as described [17].

### Assignment of parental origin

Variant specific primers were designed using the software Primer3 in combination with ClustaIW alignments. The primer sets distinguish the wild-type allele (*BRCA2 *c.8754+1-G forward: 5'-AGACCCAGCTTACCTTGA**C**G-3') from the mutation carrying allele (*BRCA2 *c.8754+1-A forward: 5'-CAGACCCAGCTTACCTTGA**A**A-3'), and the two SNP alleles in rs4942485 (rs4942485*A reverse: 5'-CTACATTACAGATGGCTAATATCTGA**T**T-3' and rs4942485*G reverse: 5'-CATTACAGATGGCTAATATCTGAAC-3'. Primer sets were tested on homozygote controls and unspecific annealing was avoided after introducing mismatches (marked in bold). PCR was carried out using standard conditions according to the manufacturer's protocols using the Expand High Fidelity^PLUS ^Taq DNA polymerase (Roche). Allele sizes were: *BRCA2 *c.8754+1-G and rs942485-G = 2297 bp, *BRCA2 *c.8754+1-*A and rs942485-G = 2298 bp, *BRCA2 *c.8754+1-G and rs942485-A = 2300 bp, *BRCA2 *c.8754+1-*A and rs942485-A = 2301 bp.

## Results and discussion

**Figure 1:**
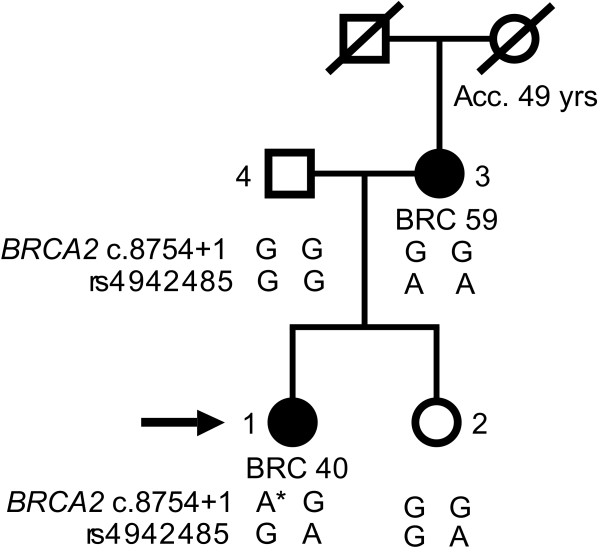
**Family pedigree**. Breast cancers are indicated as well as the age at diagnosis. Acc, accident; BRC, breast cancer. The number following the cancer gives the age at diagnosis. Moreover, the genotypes from variant specific PCR are indicated. Diagonal slash indicates deceased. The proband is indicated with an arrow. Proband = individual 1, Sister = individual 2, Mother = individual 3, Father = individual 4.

**Figure 2:**
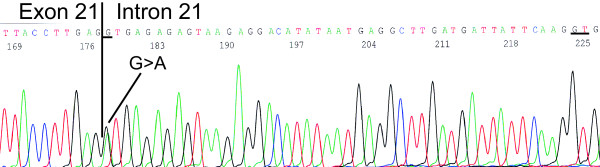
**Identification of the *BRCA2 *nucleotide 8982+1 G→A/c.8754+1 G→A variant**. DNA was purified from whole blood and *BRCA2 *exon 21 was amplified using the primers 5'-CTTTGGGTGTTTTATGCTTGT-3' and 5'-CTGGCACATCACTGAAAATC-3' and sequenced. The analysis revealed a nucleotide 8982+1 G→A/c.8754+1 G→A mutation in *BRCA2 *(sense strand). The nucleotide 8982+1 G→A/c.8754+1 G→A mutation and the cryptic splice site are underlined.

The patient was diagnosed with breast cancer at the age of 40. Since her mother had breast cancer at the age of 59 (Fig. 1), the patient was referred to genetic counselling. No other family members had breast or ovarian cancer. We analysed the entire coding region and the exon-intron boundaries of *BRCA1 *and *BRCA2 *from genomic DNA by dHPLC and sequencing and for large genomic rearrangements by MLPA analysis. The analysis revealed a polymorphism in exon 11 of *BRCA1 *(nucleotide 1186 A→G/c.1067 A→G), which is observed in approximately 5% of a control group [18], and a nucleotide 8982+1 G→A/c.8754+1 G→A mutation in *BRCA2 *that has not previously been reported in the BIC database (Fig. 2). The mutation occurs at the first base of the conserved GU splice donor site of intron 21. The mutation was verified in a second blood sample as well as in breast cancer tumour tissue.

**Figure 3:**
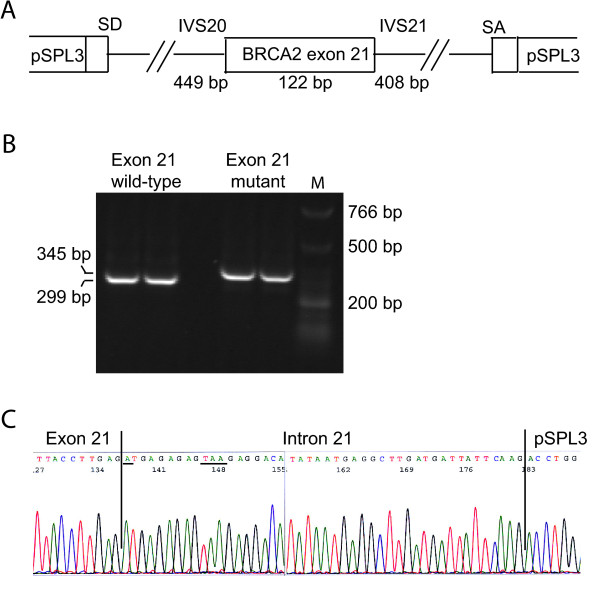
**Exon trapping analysis**. **(A) **Structure of the exon trapping vector pSPL3 containing the *BRCA2 *exon 21 and 449 bp of intron 20 and 408 bp of intron 21, respectively containing the wild-type or the nucleotide 8982+1 G→A/c.8754+1 G→A variant. **(B) **COS-7 cells were transfected with pSPL3-*BRCA2*-exon 21 wild-type or pSPL3-*BRCA2*-exon 21 mutant plasmids. Total RNA was isolated, RT-PCR analysis was performed and the PCR products (in duplicates) were resolved on a 2% agarose gel. The 299 bp product corresponds to wild-type exon 21 (unaltered splicing), while the 345 bp product corresponds to exon 21 and the inclusion of 46 bp of intron 21. The sizes of the DNA marker are indicated to the right. **(C) **Sequence of exon 21 (345 bp band). The nucleotide 8982+1 G→A/c.8754+1 G→A mutation and the TAA stop codon are underlined (sense strand).

**Figure 4:**
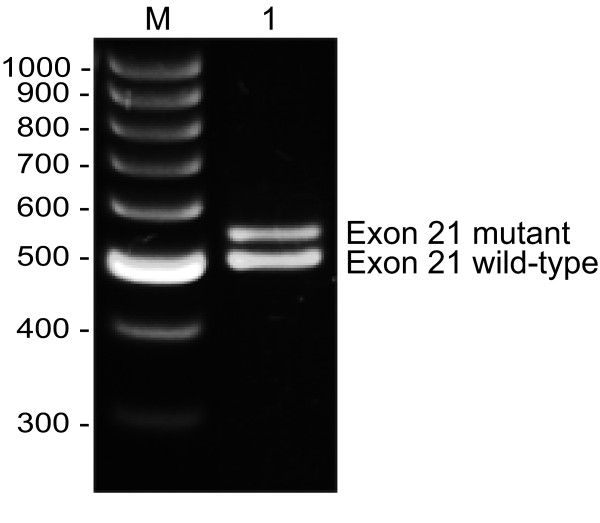
**RT-PCR was performed on RNA purified from whole blood from the proband**. The cDNA was amplified with specific *BRCA2 *primers. The sample was separated by agarose gel electrophoresis and visualized by ethidium bromide staining. Two RT-PCR products (503 bp and 549 bp) were obtained from the patient (Lane 1). The sizes of the DNA marker are indicated to the left. The PCR products were cloned and sequence analysis revealed that the 549 bp band contained the inclusion of 46 bp of intron 21 (data not shown).

To functionally characterize the *BRCA2 *nucleotide 8982+1 G→A/c.8754+1 G→A variant, a fragment containing *BRCA2 *exon 21 (122 bp), 449 bp of intron 20 (IVS20) and 408 bp of intron 21 (IVS21) containing the wild-type or the nucleotide 8982+1 G→A/c.8754+1 G→A variant, respectively, was cloned into the minigene vector pSPL3 containing exons from HIV-*tat *under the control of the SV40 promoter (Fig. 3A) [19]. Constructs containing either the wild-type or the mutant IVS21 sequence was transfected into COS-7 cells. After 48 hours mRNA was purified and examined by RT-PCR. The splicing products were separated on a 2% agarose gel. The wild-type construct yields a product of 299 bp and a product of 177 bp if exon 21 is excluded from the transcript (exon skipping). Moreover a larger product than 299 bp would demonstrate that additional intron sequence is included in the transcript. The normal wild-type *BRCA2 *exon 21 (pSPL3-*BRCA2*-wt) generated one transcript comprising the expected 299 bp, while the *BRCA2 *nucleotide 8982+1 G→A/c.8754+1 G→A mutant (pSPL3-*BRCA2*-mut) yielded a band with slightly higher mobility (Fig. 3B). Sequencing revealed, that it contained an additional 46 bp from intron 21 generating a transcript of 345 bp (Fig. 3C), indicating that it activates a cryptic splice site following these 46 bp (Fig. 1). To provide direct evidence for the aberrant splicing, RNA was isolated from whole blood of the affected patient and RT-PCR was performed before the products were analysed on a 1% agarose gel (Fig. 4). Two PCR products – one with the expected size of the wild-type (503 bp) and an additional RT-PCR product (549 bp) – were amplified from the patient. Cloning and sequence analysis of the latter verified the inclusion of 46 bp of intron 21 (data not shown).

We examined the patient's mother, who was affected with the same type of breast cancer at the age of 59 years, for the same mutation, but she did not have this mutation in either DNA purified from blood or breast cancer tumour samples. Therefore mosaicism was excluded. Moreover, the father and the patient's sister did not carry the mutation. To establish the paternity we performed a SNP microarray analysis using 250K Sty gene array (Affymetrix). The analysis provided the relatedness estimates (Table 1), where k0 is the probability that pairs of individuals at a random loci share no allele identical by descent (IBD), k1 is the probability that pairs of individuals at a random loci share one allele IBD, and k2 the probability that pairs of individuals at a random loci share two allele IBD. The expected relatedness for full siblings is k0 = 0.25, k1 = 0.5, k2 = 0.25, for parent offspring k0 = 0, k1 = 1, k2 = 0, for unrelated k0 = 1, k1 = 0, k2 = 0, which in all cases fits in this family. We therefore conclude that the identified mutation in the proband is a *de novo *mutation in *BRCA2*. In contrast to the previously identified *de novo BRCA2 *nucleotide 3034del4 and *BRCA1 *nucleotide 3888delGA mutations [14,15], the mutation reported in our study resides in a new position not previously reported in the BIC database. This was also the case with the *BRCA2 *nucleotide 7260insA mutation [13]. Several other variants have been detected in the vicinity of the splice donor site in exon 21, including nucleotide 8982 G→A/c.8754 G→A (reported once), nucleotide 8982+1 A→G/c.8754+4 A→G (reported seven times), nucleotide 8982+5 G→A/c.8754+5 G→A (reported once) and nucleotide 8982+5 G→T/c.8754+5 G→T (reported once), suggesting that this region could be prone to mutations.

**Table 1:** Relatedness estimates.

**Individual**	**Individual**	**k0**	**k1**	**k2**	**Relationship**
1	2	0.23	0.49	0.28	full siblings
1	3	0.01	0.99	0.00	parent offspring
1	4	0.01	0.99	0.00	parent offspring
2	3	0.01	0.98	0.01	parent offspring
2	4	0.01	0.99	0.01	parent offspring
3	4	0.98	0.02	0.00	unrelated

Relationship was infered by estimating the pairwise *identical by descent *(IBD) sharing as described in [17]. k0 is the probality that pairs of individuals at a random loci share no allele IBD, k1 the probality that pairs of individuals at a random loci share one allele IBD, and k2 the probality that pairs of individuals at a random loci share two allele IBD. The expected relatedness for full siblings is k0 = 0.25, k1 = 0.5, k2 = 0.25, for parent offspring k0 = 0, k1 = 1, k2 = 0, and for unrelated k0 = 1, k1 = 0, k2 = 0. The individual number is indicated in figure 1.

To determine the parental origin of the mutation, variant specific PCR was performed using primers designed to distinguish between two SNP alleles and the mutation carrying allele from the wild-type allele. The father was homozygote for the haplotype GG, while the mother was homozygote for the haplotype GA (see Fig. 1). PCR analysis of the proband revealed a band using mutant *BRCA2 *and rs4942485*G primers, indicating that the haplotype *AG must derive from the father, and that the mutation therefore arose in the testicular germ cells changing *BRCA2 *nucleotide 8982+1/c.8754+1 from G to A. This is in agreement with previous findings in *BRCA1 *[14], whereas the studies describing *de novo *mutations in *BRCA2 *were unable to determine parental origin [13,15]. Examinations of other cancer families, including MEN2B and retinoblastoma families have indicated that *de novo *mutations primarily occur in the male germ line [20,21] and that the fathers age is a major determinant since mutations accumulate during life [22]. It is, however, noteworthy that the age of the probands parents in our study was only 19 and 21 years at the time of birth of their affected daughter.

## Conclusion

We conclude that the *BRCA2 *nucleotide 8982+1 G→A/c.8754+1 G→A mutation is a *de novo *mutation arising from the male germ line. The mutation is not previously reported in the BIC database. It leads to the activation of a cryptic splice site 46 base pairs 3' of exon 21 and introduces a premature stop codon and thereby a truncated BRCA2 protein. Therefore, this mutation can be classified as a disease-causing mutation. Analysis of intronic *BRCA1 *and *BRCA2 *variants by functional splicing assays can provide information that can be used clinically.

## Competing interests

The authors declare that they have no competing interests.

## Authors' contributions

TVOH designed the study, supervised exon trapping, interpreted the results, and wrote the draft of the paper. MLB was involved in the designing of the study, the variant specific PCR, and the writing of the manuscript. LJ performed the RT-PCR analysis on RNA from whole blood and was involved in the writing of the manuscript. AA performed the relationship SNP analysis. BF–B analysed the carcinoma samples. HE designed and performed the variant specific PCR. BE selected the patients and participated in genetic counseling and was involved in the writing of the manuscript. FCN designed the study and was involved in the writing of the manuscript. All authors have read and approved the final manuscript.

## Pre-publication history

The pre-publication history for this paper can be accessed here:


